# Laser-ultrasonic delivery of agents into articular cartilage

**DOI:** 10.1038/s41598-017-04293-5

**Published:** 2017-06-21

**Authors:** Heikki J. Nieminen, Gonçalo Barreto, Mikko A. Finnilä, Alejandro García-Pérez, Ari Salmi, Sanjeev Ranjan, Kari K. Eklund, Kenneth P. H. Pritzker, Simo Saarakkala, Edward Hæggström

**Affiliations:** 1 Electronics Research Laboratory, Department of Physics, University of Helsinki, Helsinki, Finland; 2 Research Group of Medical Imaging, Physics and Technology, Faculty of Medicine, University of Oulu, Oulu, Finland; 3 Department of Laboratory Medicine and Pathobiology, University of Toronto, Toronto, Canada; 4 Orton Orthopaedic Hospital and Research Institute, Invalid Foundation, Helsinki, Finland; 5 Department of Medicine, University of Helsinki, Helsinki, Finland; 6 Department of Applied Physics, University of Eastern Finland, Kuopio, Finland; 7 Department of Electronic Engineering, Higher Technological Institute of Poza Rica, Poza Rica, México USA; 8 Laboratory of Radiochemistry, Department of Chemistry, University of Helsinki, Helsinki, Finland; 9 Department of Rheumatology, University of Helsinki and Helsinki University Hospital, Helsinki, Finland; 10 Department of Laboratory Medicine and Pathobiology, Mount Sinai Hospital, Toronto, Canada; 11 Department of Diagnostic Radiology, Oulu University Hospital, Oulu, Finland

## Abstract

Research is ongoing to develop drug therapies to manage osteoarthritis (OA) and articular cartilage (AC) injuries. However, means to deliver drug to localized AC lesions are highly limited and not clinically available. This study investigates the capability of laser ultrasound (laser-induced plasma sound source) to deliver agents (methylene blue, MB, in PBS) into bovine AC. Treatment samples (*n* = 10) were immersed in MB solution simultaneously with LU exposure, while adjacent control 1 tissue (*n* = 10) was pre-treated with LU followed by immersion in MB and adjacent control 2 tissue (*n* = 10) was only immersed in MB. AC exposed (*n* = 22) or not exposed (*n* = 27) to LU were characterized for anomalies in structure, composition, viability or RNA expression. Optically detected MB content was significantly (*p* < 0.01) higher in treatment samples up to a depth of 500 µm from AC surface as compared to controls. No major unwanted short-term effects on AC structure, proteoglycan or collagen contents, chondrocyte viability or RNA expression levels were detected. In conclusion, LU can deliver agents into AC without major short-term concerns on safety. LU could reveal new strategies for the development of localized drug therapies in AC.

## Introduction

Osteoarthritis (OA) can be considered as a group of joint diseases; the pathogenesis is characterized by regenerative, reparative, and degenerative structural changes in all tissues of the joint, including the articular cartilage (AC), bone, synovium, capsule and periarticular soft tissues^1, 2^. Upon OA progression, AC is degenerated often with local lesions^3^. Even in normal AC, accidents can induce AC trauma that can be very focal and yet develop into post-traumatic OA. Advanced AC degeneration causes substantial pain, impairment of mechanical function^2^ and, eventually, can lead to bone-to-bone contact and disability of the diseased joint. Because curative treatment for OA is still to be discovered, there is a major interest to develop means to slow down, halt, and even reverse OA progression.

Several disease-modifying OA drugs (DMOADs) are under development^4–6^. A key goal in these drug therapies is to affect AC morphology and restore the mechanical function of the joint^7^. For instance diacerein (~368 Da) and glucosamine (~179 Da) have shown promise in AC regeneration. However, candidate DMOADs administered orally may have insufficient residence time in the joint to diffuse into AC, because avascularity and unique and dense composition of the tissue make AC resistant to drug access. Growth factors have proven regenerative effects in AC, but they may induce unwanted effects outside the lesion; *e.g*. TGFβ can promote AC regeneration, but it can induce osteophytes and cause inflammation^8, 9^. Physically forcing drug agents locally into AC lesions would (i) permit development of targeted and personalized OA therapy, (ii) enhance safety by preventing unnecessary drug exposure to adjacent tissue and the rest of the body, and (iii) increase the concentration and residence time of drug molecules within the target. Unfortunately, physical techniques to locally and non-destructively deliver drugs into AC lesions are not yet clinically available.

Ultrasound is commonly associated with tissue characterization^10, 11^ and imaging^12, 13^. However, extensive literature exists on the potential of high-intensity ultrasound (HIU) in drug delivery applications, *e.g*. delivery of entities into skin^14^, releasing drugs from micro-capsules inside tumor^15^, delivery of genes into cells^16^ and opening of the blood-brain-barrier brain for enhanced drug penetration^17^. In a broader context, the potential of HIU to contribute to drug delivery relates to its capability to manipulate material; *e.g*. HIU can (i) translate tissue, particles or voids, (ii) induce fluid and ionic streams, (iii) modify tissue or cell membrane permeability and (iv) generate controlled thermal effects^18^.

We demonstrated earlier that kHz and MHz ultrasound can deliver molecules and particles into AC^19–21^. However, kHz HIU may damage AC through a phenomenon called transient cavitation^19, 22^. Furthermore, MHz delivery of kDa-sized molecules did not demonstrate tissue damage, but is time-consuming (2.5 hrs to deliver up to half-way into the AC)^21^. Our early experiments suggested that laser-induced ultrasound pulses may contribute to localized delivery of agents into AC within a time frame of minutes^20^. In the present study, we aim (i) to quantify the delivery of agents into AC by LU and (ii) to conduct an initial evaluation on whether major short-term safety effects are induced by LU.

## Results

**Figure 1:**
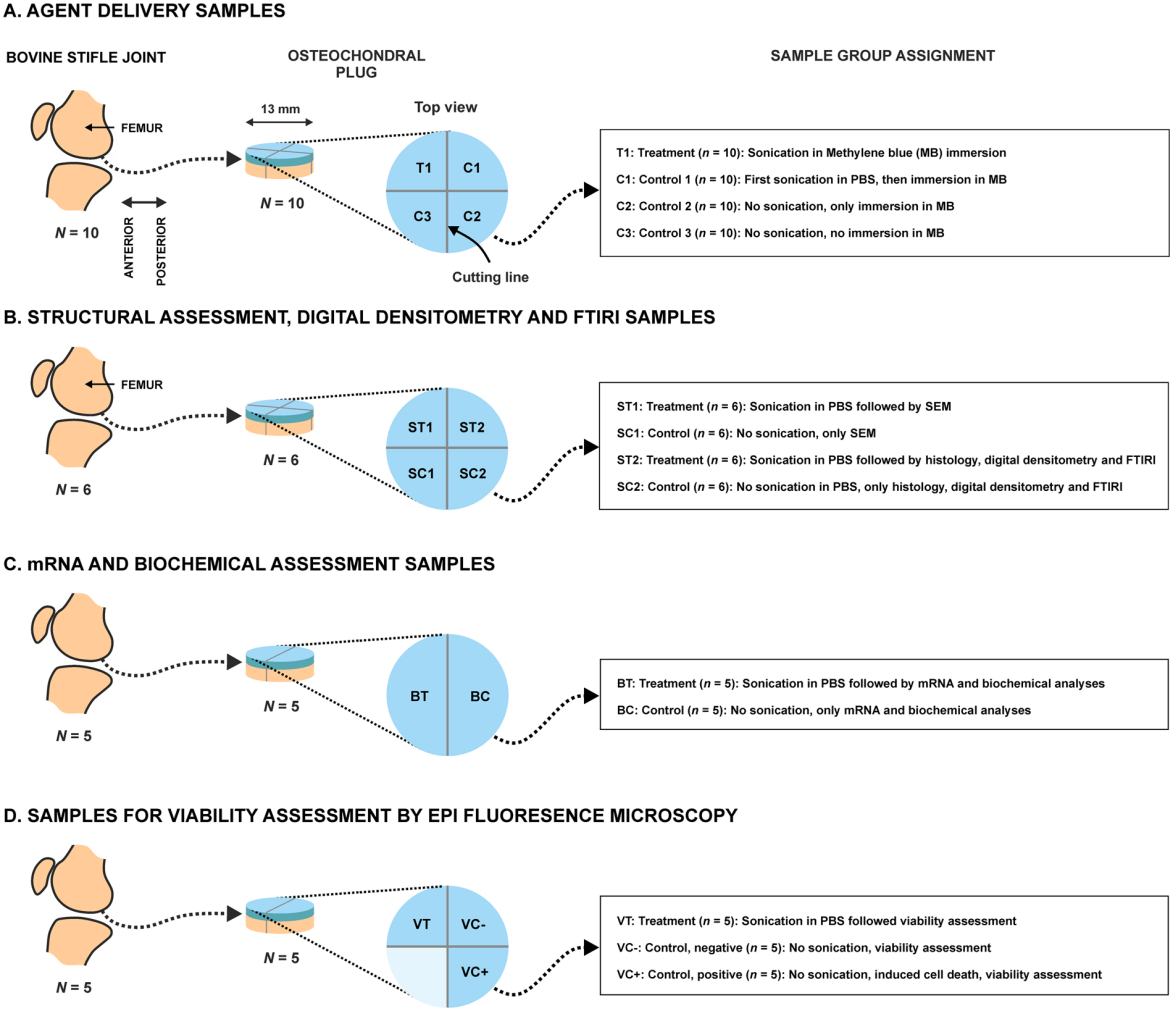
(**A**) Preparation and group assignment of agent delivery samples: Osteochondral plugs (*N* = 10, Ø = 13 mm) were prepared from bovine stifle joints (one plug per joint, one joint per animal). The plug was then cut into four quadrants and randomized to the treatment group (T1) or control group (C1, C2 or C3). (**B**) Preparation and group assignment of structural assessment, digital densitometry and Fourier transform infrared imaging (FTIRI) samples: Osteochondral plugs (*N* = 6, Ø = 13 mm) were prepared from bovine stifle joints (one plug per joint, one joint per animal). The plug was then cut into four quadrants and randomized to the treatment group (ST1 or ST2) or control group (SC1 or SC2). (**C**) Preparation and group assignment of mRNA and biochemical assessment samples: Osteochondral plugs (*N* = 5, Ø = 13 mm) were prepared from bovine stifle joints (one plug per joint, one joint per animal). The plug was then cut into two halves and randomized to the treatment group (BT) or control group (BC). (**D**) Samples for viability assessment by EPI fluorescence microscopy: Osteochondral plugs (*N* = 5, Ø = 13 mm) were prepared from bovine stifle joints (one plug per joint, one joint per animal). The plug was then cut into four quadrants. Three quadrants were randomized to the treatment group (VT), negative control group (VC−) or positive control group (VC+). One quadrant remained unused.

**Figure 2:**
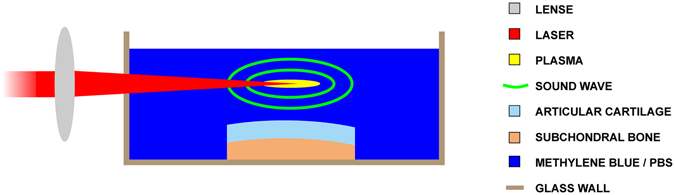
Experimental setup. Articular cartilage (AC) sample with subchondral bone was placed into a glass container filled with immersion fluid (methylene blue in PBS or PBS only). Laser beam (λ = 1064 nm) was focused 3 mm above the AC surface. Ultrasound pulse was generated from a minute plasma spark at the optical focus following a 8 ns laser pulse (Q-switch delay = 300 µs, pulse energy = 130 ± 10 mJ). One treatment consisted of 2000 sound pulses at 3 Hz pulse repetition frequency yielding a treatment time of 11.1 min.

**Figure 3:**
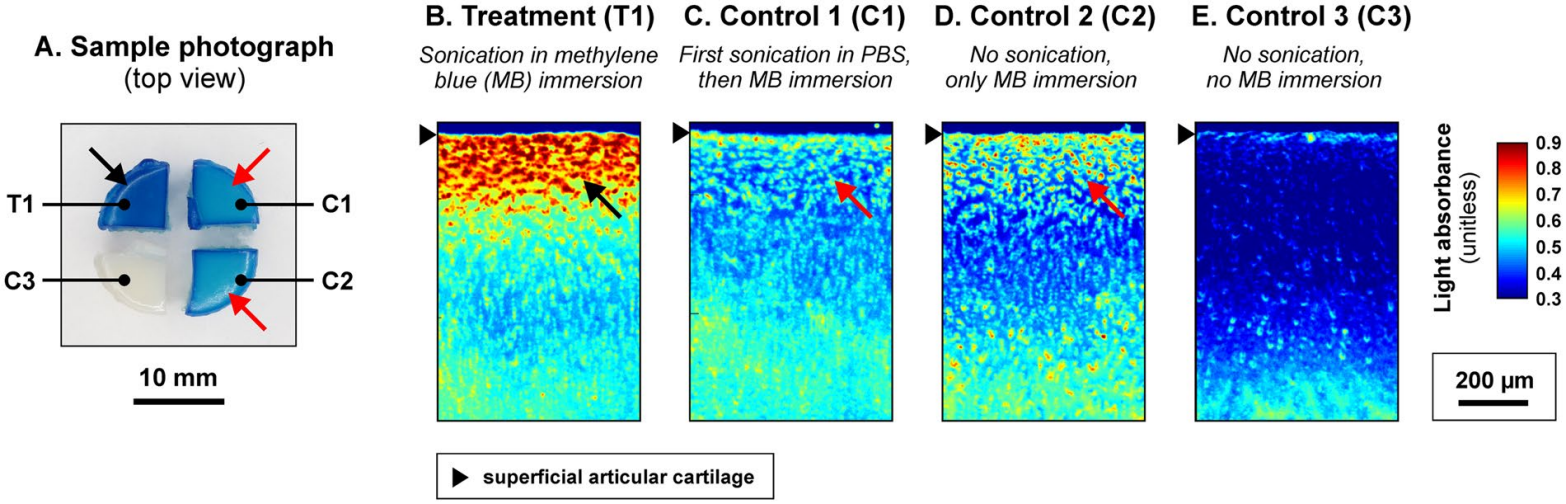
Photographs of treated sample quadrants (**A**) and optical absorbance images of articular cartilage (AC) samples (**B**–**E**) exposed to methylene blue (MB). The blue contrast in photograph and absorbance in AC sections were greater in T1 (black arrows) as compared to C1 and C2 (red arrows). No difference in absorbance was observed between C1 to C2 suggesting that ultrasound treatment does not modify permeability of AC.

**Figure 4:**
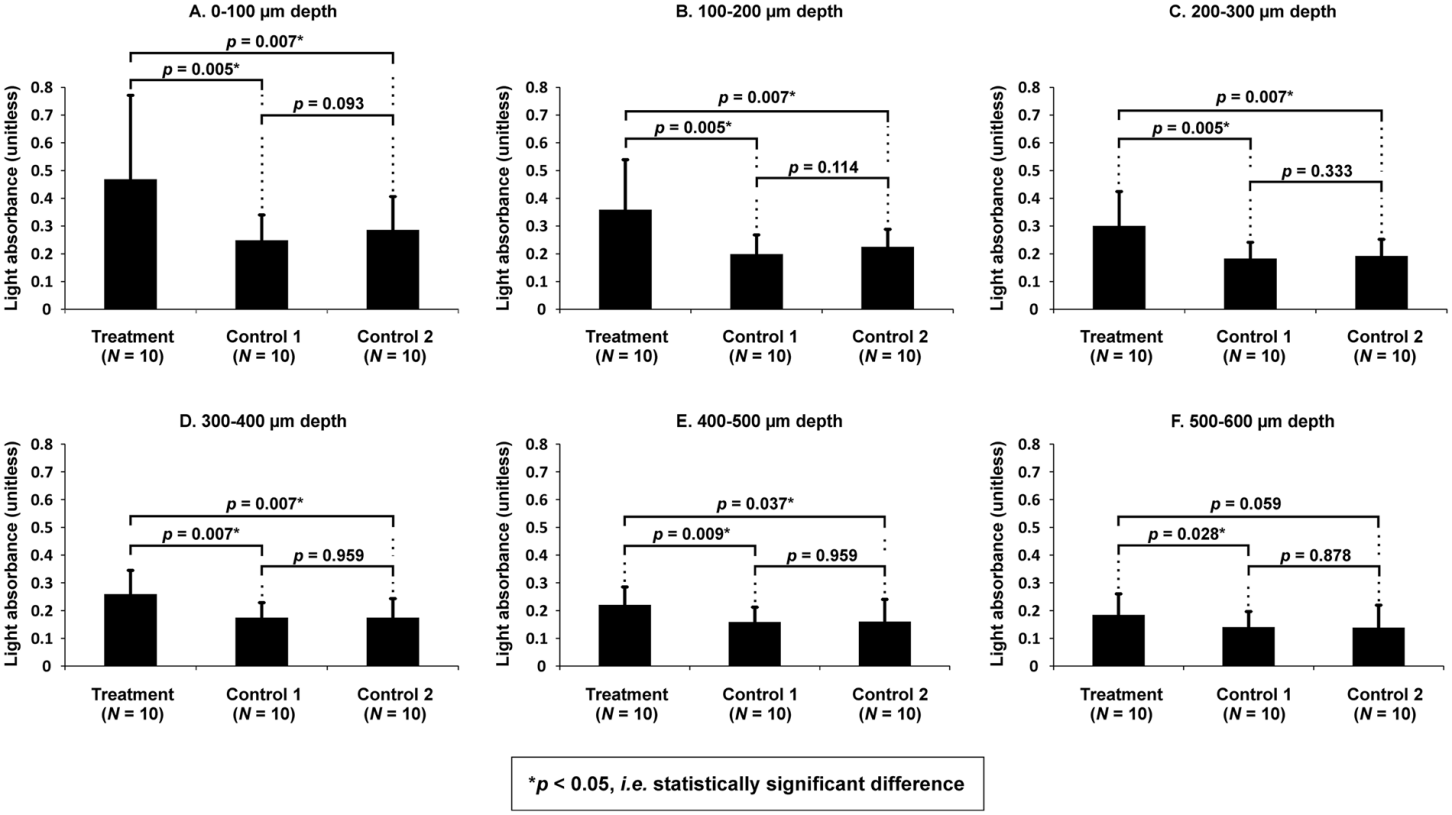
Depth-wise optical absorbance (mean ± 95% CI) in different sample groups. Absorbance, representing methylene blue (MB) content, was statistically significantly greater up to a depth of 400–500 µm in T1 (ultrasound exposure during MB immersion) compared to C1 (ultrasound exposure in PBS followed by MB immersion). Absorbance was greater in T1 up to a depth of 500–600 µm compared to C2 (no ultrasound treatment, only MB immersion). No significant (p > 0.05; Friedman followed by Wilcoxon) difference between groups was observed at depths > 600 µm from AC surface. The result suggests that laser-ultrasound can deliver MB into AC.

First we studied, using three experimental groups T1, C1 and C2 (Fig. 1, Table 1), whether LU (Fig. 2, *f*_c_ = 3.0 ± 0.1 MHz; bandwidth at −3 dB = 2.5 ± 0.1 MHz; peak-positive-pressure = 9.14 ± 0.15 MPa; peak-negative-pressure = 1.90 ± 0.05 MPa; *I*_TP_ = 6017 ± 188 W/cm^2^; *I*_PA_ = 1076 ± 90 W/cm^2^; *I*_TA_ = 1.86 ± 0.04 mW/cm^2^; mechanical index = 1.01 ± 0.03; mean ± S.E.M, *n* = 100; parameters were defined with a needle hydrophone in ion-exchanged water at 3 mm distance from the plasma spark, which is equivalent to AC surface location) could enhance the delivery of MB into AC. Optical absorbance, an indicator of MB content in AC (Supplementary Figure A1), was statistically significantly (*p* < 0.01 or *p* < 0.05) different in LU exposure group T1 (*n* = 10) as compared to the control C1 (*n* = 10) at all AC depths up to 600 µm (Figs 3 and 4). The absorbance was different (*p* < 0.01 or *p* < 0.05) in T1 compared to C2 (Table 1) (*n* = 10) up to a depth 500 µm (Figs 3 and 4). The absorbance in control groups C1 and C2 were not statistically different at any depth (*p* > 0.05) (Figs 3 and 4).

**Table 1:** Summary of experimental procedures by sample group. The procedures for each sample were applied chronologically from the far left column towards far-right column.

Sample	LU exposure during PBS immersion	LU exposure during MB immersion	Immersion in MB	Characterizing technique
Treatment, T1 (*n* = 10)	No	Yes	No	LM
Control 1, C1 (*n* = 10)	Yes	No	Yes	LM
Control 2, C2 (*n* = 10)	No	No	Yes	LM
Control 3, C1 (*n* = 10)	No	No	No	LM
Structural assessment, treatment 1, ST1 (*n* = 6)	Yes	No	No	FESEM
Structural assessment, control 1, SC1 (*n* = 6)	No	No	No	FESEM
Structural assessment, digital densitometry and FTIRI, treatment 2, ST2 (*n* = 6)	Yes	No	No	SH, DD, FTIRI
Structural assessment, digital densitometry and FTIRI, control 2, SC2 (*n* = 6)	No	No	No	SH, DD, FTIRI
mRNA and biochemical assessment, treatment, BT (*n* = 5)	Yes	No	No	mRNA, Bioch.
mRNA and biochemical assessment, control, BT (*n* = 5)	No	No	No	mRNA, Bioch.
Viability assessment, treatment, VT	Yes	No	No	EPI-FM
Viability assessment, negative control, VC−	No	No	No	EPI-FM
Viability assessment, positive control, VC+	No	No	No	EPI-FM

LU = laser-ultrasound.

PBS = phosphate-buffered saline.

MB = methylene blue (0.005% w/v) in PBS.

LM = light microscopy in transmission mode at peak wavelength 657 nm.

EPI-FM = EPI fluorescence microscopy.

FESEM = field emission scanning electron microscopy.

FTIRI = Fourier transform infrared imaging (Amide I absorption, collagen).

SH = standard histology with Safranin O -staining (proteoglycan).

DD = digital densitometry for assessment of fixed-charge density in Safranin O –stained sections.

mRNA = mRNA analysis.

Bioch. = biochemical analysis.

**Figure 5:**
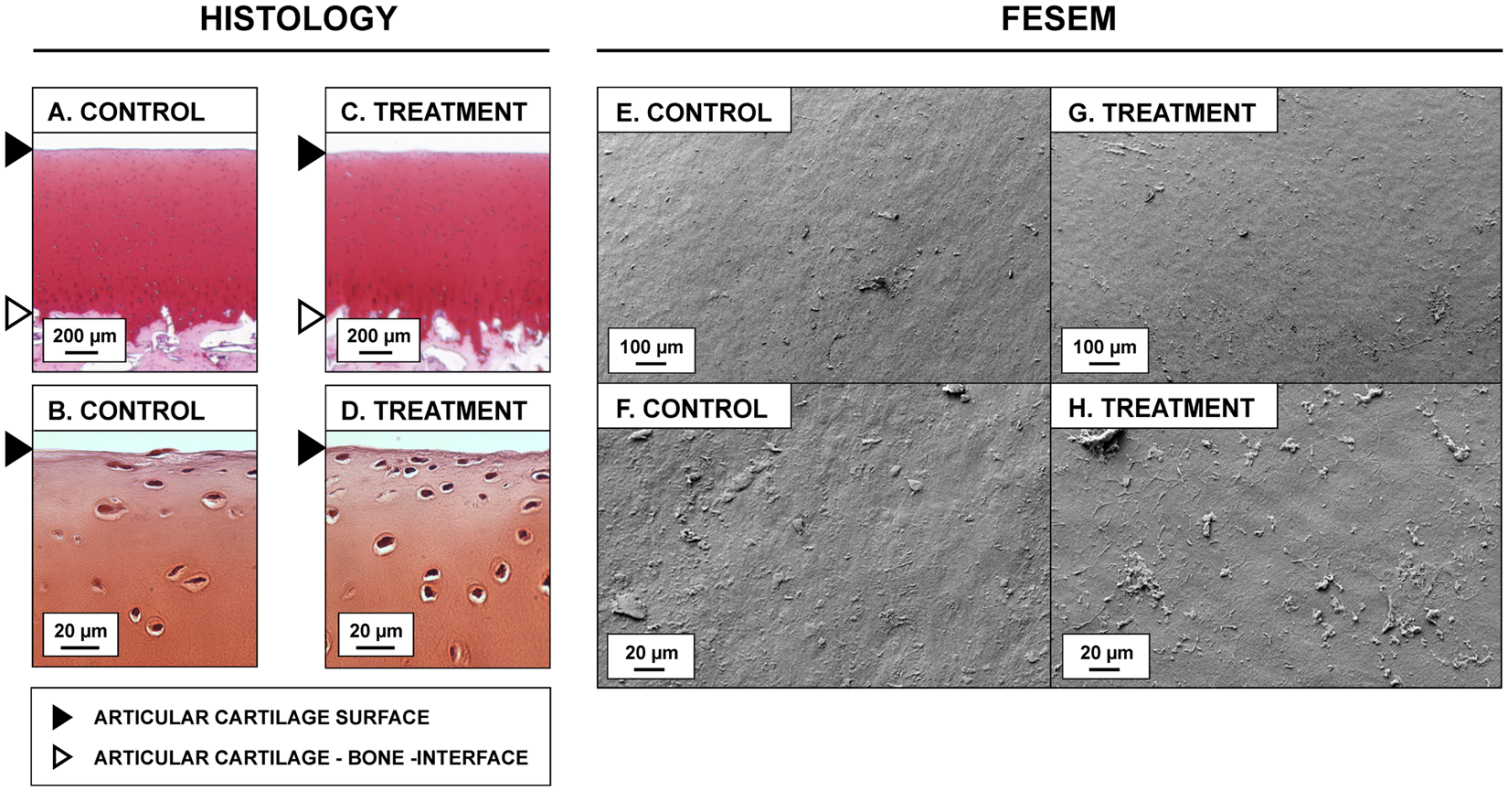
Damage assessment. No differences in superficial fibrillation or in fixed charge density (red contrast, binds stoichiometrically to proteoglycan) were observed in safranin O -stained articular cartilage (AC) control sections (**A**,**B**), Control, (*n* = 6) as compared to sections from adjacent AC tissue exposed to laser-ultrasound (LU) (**C**,**D**), treatment, (*n* = 6). Similarly, field emission scanning electron imaging (FESEM) assessment did reveal no structural damage to the AC surface when comparing control samples (**E**,**F**), (*n* = 6) to treatment samples (**G**,**H**), (*n* = 6). These results suggest that LU applied in this study does neither cause structural damage to AC surface, which is rich in collagen, nor induce proteoglycan depletion.

**Figure 6:**
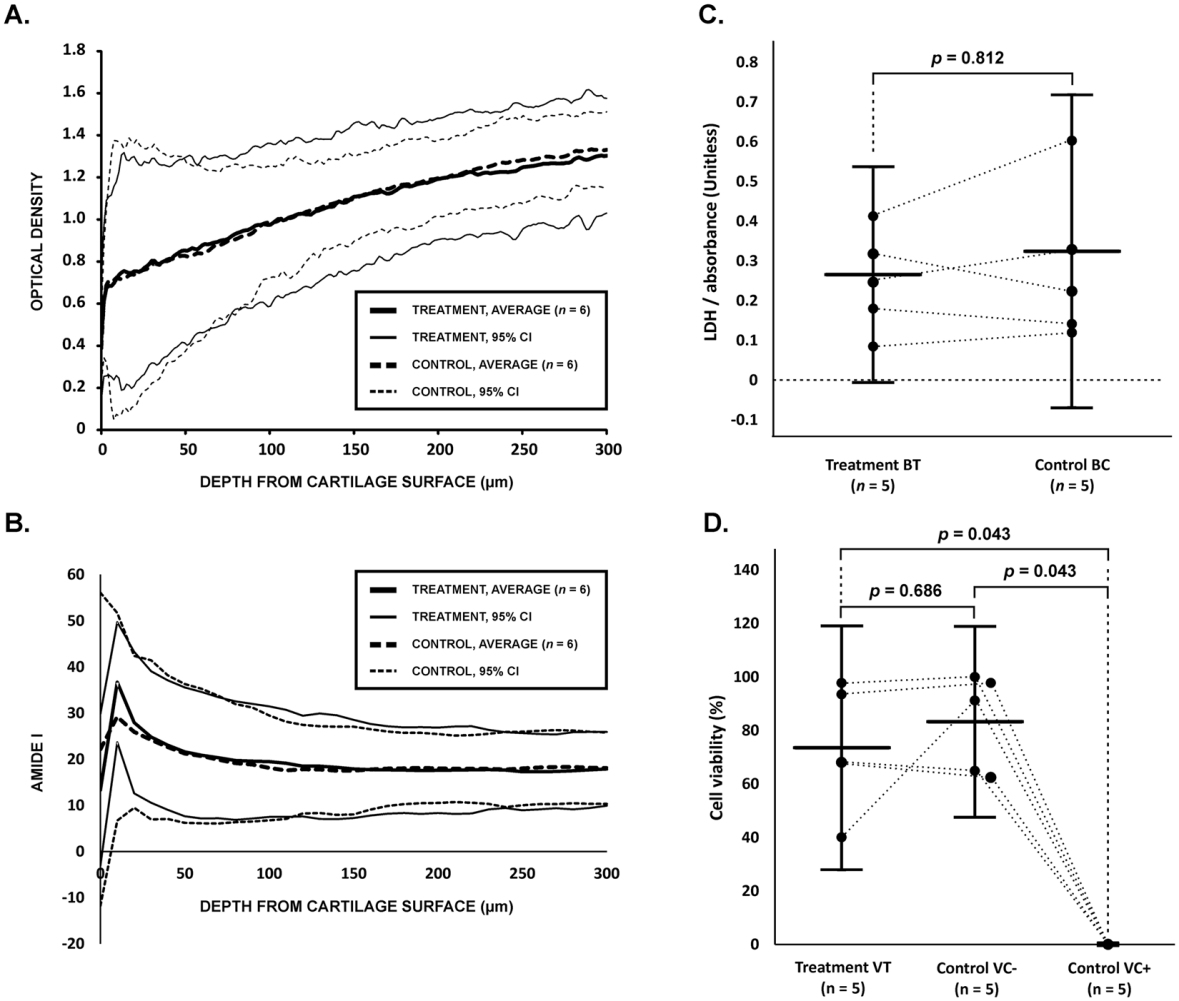
(**A**) Depth-wise optical density (mean ± 95% CI) of safranin O -stained articular cartilage (AC) sections detected with digital densitometry. The AC subjected to laser-ultrasonic (LU) exposure has a similar depth-wise optical density profile as the adjacent control tissue. The result suggests that no proteoglycan loss is induced by LU exposure. The analysis was applied to AC surface (0–300 µm) since leakage of proteoglycans, if present, would be expected to occur in superficial AC. (**B**) Depth-wise amide I peak absorbance (mean ± 95% CI, arbitrary units) of non-stained articular cartilage (AC) sections detected with Fourier transform infrared image imaging. The result suggests that no collagen loss is induced by LU exposure. The analysis was applied to AC surface (0–300 µm). (**C**) Content of lactate dehydrogenase (LDH) was optically quantified as absorbance in the supernatant 24 h post LU and compared with adjacent controls not exposed to LU. The difference was statistically insignificant (p = 0.812) suggesting no cell death. (**D**) Cell viability assessment of AC stained with dead/live cell kit and EPI fluorescence microscopy demonstrate that cell viability was chondrocyte was not different (p = 686) in AC exposed to LU compared to negative control samples VC−. This result is in line with LDH experiments suggesting no immediate chondrocyte death due to LU. All statistics (**A**–**C**) are presented as mean ± 95% CI. The p-values < 0.05 were considered statistically significant.

We assessed the effect of LU on the morphology and viability of AC. In field emission scanning electron microscopy (FESEM) images, no difference in morphology of superficial AC was observed in ST1 (*n* = 6) as compared to SC1 (*n* = 6) (Figs 1 and 5, Table 1). LM images of Safranin O –stained sections demonstrated no difference at any depth in Safranin O red contrast (indicator for fixed-charge density) or qualitative differences in AC matrix or chondrocytes when comparing ST2 (*n* = 6) and SC2 (*n* = 6) (Figs 1 and 5, Table 1). In digital densitometry (DD), depth-wise DD profiles of Safranin-O –stained sections of ST2 and SC2 showed no differences (Fig. 6A), which suggests no proteoglycan loss. In Fourier transform infrared imaging (FTIRI), depth-wise profiles of Amide I peak absorbance of unstained sections of ST2 and SC2 showed no differences (Fig. 6B), which suggests no collagen loss. Cell viability measured by 24 h release of LDH remained unchanged between BT (*N = 5*) and BC (*N* = 5) (Table 1) AC explants groups (*p* = 0.812) suggesting that cell viability was not significantly influenced by LU exposure during the experimental time period (Fig. 6C). As detected from EPI fluorescence microscopy cell viability in LU-treated samples (VT) did not differ from negative (untreated) control VC- (*p* = 0.686), but was significantly different than viability of positive (thermally damaged) control VC+ (*p* = 0.043) (Fig. 6D).

**Figure 7:**
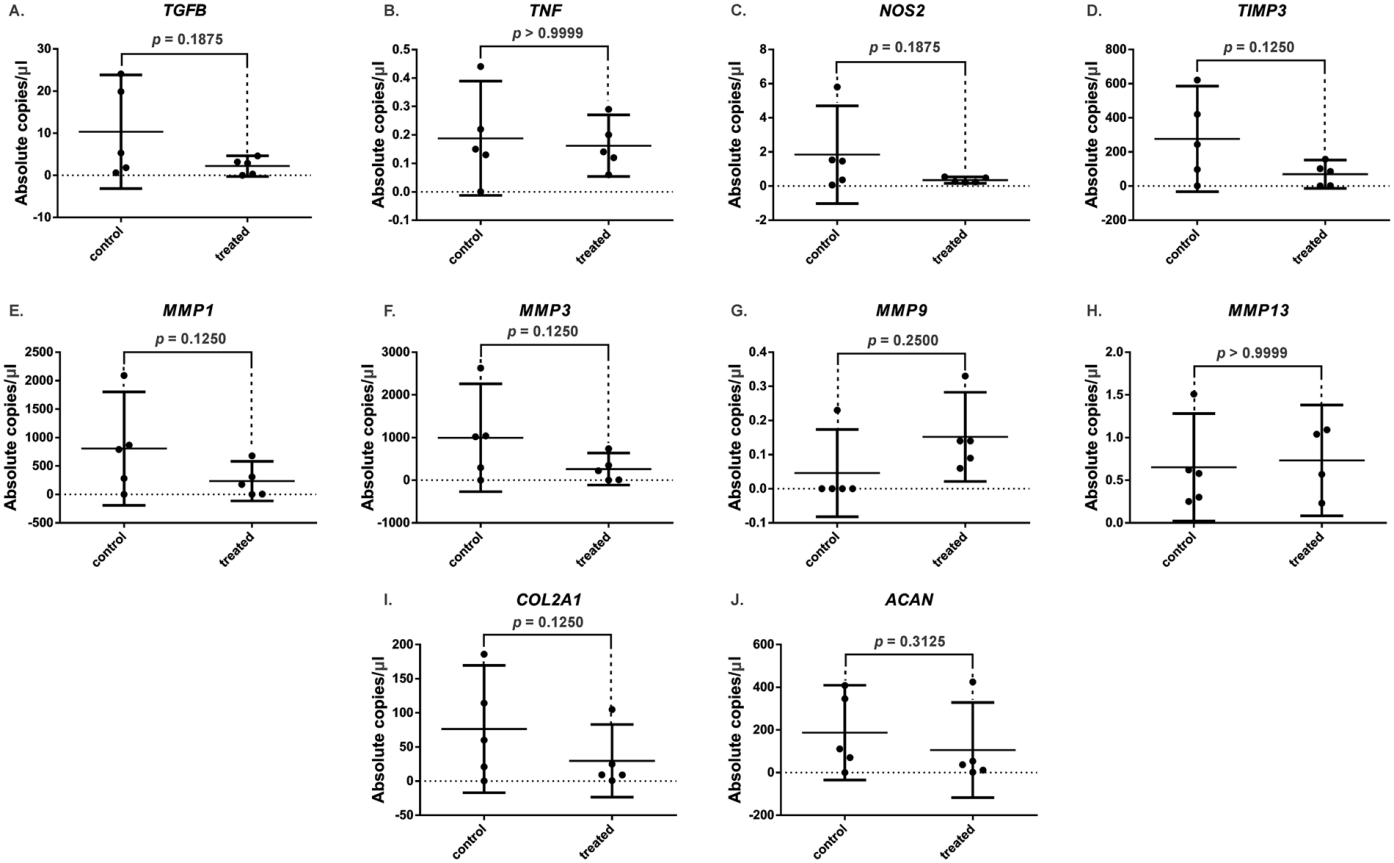
Effect of LU on the chondrocyte expression of catabolic, inflammatory and anabolic markers dysregulated in OA. Absolute copies concentration levels of corresponding genes per μl of PCR reaction measured by digital PCR assay. Gene expression of the studied gene mRNA levels remained mostly unaltered under LU exposure (*p* > 0.100, Wilcoxon) (individual biological replicates are shown (*n* = 5 per group) as well as mean ± 95% confidence intervals; samples were measured as technical duplicates and averaged). The results indicate that interaction between LU pulses and chondrocyte may be a promising anti-catabolic approach to be further explored in the inflammatory management of OA.

AC degeneration and OA progression is associated with upregulation of catabolic factors; therefore, we studied whether LU had any effect on the baseline mRNA expression levels. LU did not alter significantly *TGFB, TNF, NOS2, TIMP3, MMP1, MMP3, MMP9*, or *MMP13* mRNA expression levels in healthy AC, when compared to AC not exposed LU (Fig. 7). Moreover, mRNA expression levels of *COL2A1* and *ACAN*, the essential molecules of AC structure, were not significantly altered.

## Discussion

We investigated whether laser-induced ultrasound pulses can deliver MB into bovine AC. We applied FESEM, histology, DD and FTIRI to reveal whether LU sonication could have damaging effects on AC. In addition, chondrocyte gene expression levels and cell viability were also analyzed.

The optical absorbance at 657 nm was up to a depth of 500–600 µm statistically greater in treated samples than in controls from adjacent tissue. The absorbance in controls 1 and 2 did not differ at any depth. Therefore, the results suggest that LU delivers MB into AC when both sound and delivered entity are simultaneously present. As the LU treatment took about 11 min, the timeframe could be clinically practical.

Several potential explanations for delivery mechanisms exist. These include acoustic streaming and radiation force, temporary/long-term modification of permeability, or heat or cavitation –induced damage, some transport mechanisms being more probable than others. LU generated with plasma sparks produces short and broadband ultrasound pulses with high pressure. The sound can, therefore, produce pulsating acoustic streaming (due to sound absorption) in the fluid phase^23^ and acoustic radiation forces that can palpate the fluid-AC interface and AC tissue^24^. This is in line with our observation that the immersion fluid surface vibrated during the LU sonication. Actuating the fluid phase near AC the surface with streams of fresh MB would maximize concentration difference between outside and inside of AC according to Fick’s first law^25^ and, thus, could enhance molecular flux towards the deep AC^26^. In addition, when interacting with micro/nano-bubbles, the ultrasound could generate fluid movement around the bubbles^27^ or tissue constructs (*e.g*. collagen bundles), which could contribute to MB convection and, therefore, could enhance molecule flux towards the deep tissue. While evidence on momentary modification of AC permeability cannot be proved with the current data, it is still one possible mechanism, since the radiation force palpating the tissue could modify the size and shape of nano-cavities within tissue. Since there was no statistical difference (*p* > 0.05) in the optical absorbance between C1 and C2, the MB delivery cannot be explained by damage or long-term modification of AC permeability. Apparent absence of structural damage to AC is also supported by the structural assessment experiments: LU-treated and adjacent control tissue appeared similar in FESEM, histology, and DD. No depletion of proteoglycans or collagen, the main constituents of the AC solid matrix^28^, were identified based on DD or FTIRI assessments, respectively. AC surface, rich in collagen, was not impacted as observed in FESEM. Moreover, leakage of proteoglycan would have been expected if collagen structure would have disrupted^28^. Therefore, the results suggest that no short-term damage of proteoglycans or collagen occurred. While increasing temperature can increase diffusion in human AC^29, 30^, on average the temperature of the immersion fluid never rose more than 1 °C and, therefore, thermal mechanisms are unlikely to contribute to thermal damage or to explain the observed delivery.

Authors have previously demonstrated delivery agents into articular cartilage (AC) using MHz high-intensity focused ultrasound (HIFU)^21^ (frequency = 1.138 MHz, PPP = 2.70 ± 0.06 MPa, PNP = 1.18 ± 0.01 MPa; *I*_sptp_ = 488 W/cm^2^, *I*_sppa_ = 102 W/cm^2^, *I*_spta_ = 5 W/cm^2^; pulse repetition frequency = 285 Hz; cycles per pulse = 200; mechanical index 1.1). In this study the LU intensities were *I*_TP_ = 6 017 ± 188 W/cm^2^; *I*_PA_ = 1 076 ± 90 W/cm^2^; *I*_TA_ = 1.86 ± 0.04 mW/cm^2^. Therefore, the pulse-average and time-averaged radiation pressures were 680 Pa and 33 Pa in MHz HIFU and 7.3 kPa and 13 mPa in LU, respectively. The PRF and time-averaged radiation pressure in LU were low compared to those in MHz HIFU. On the other hand the radiation pressure during a pulse in LU is >10x than in MHz HIFU. Thus, it is likely that the delivery mechanisms are associated with the transient LU pulses rather than with the time-averaged effects. This would be expected to palpate the AC and generate liquid streams in MB at a low repetition rate of 3 Hz. On the contrary, in the MHz HIFU, the pulse averaged radiation pressure was lower (680 Pa) and time-averaged radiation pressure was greater (33 Pa). This US exposure would be expected to induce a lower pressure palpation of AC at a relatively high repetition rate (200 Hz). Therefore, in the liquid phase, the flow should be relatively constant.

In both, HIFU^21^ and LU of present study, the mechanical indices are rather low, 1.1 and 1.0, respectively. This suggests that both approaches are similar in terms of low risk for deleterious cavitation. The low time-averaged intensity of LU (1.86 mW/cm^2^) suggests that the thermal safety is greatly enhanced by LU since in MHz HIFU approach the spatial peak time-averaged intensity (5 W/cm^2^) was >1000x higher. With MHz HIFU, the delivery of ~2.9 kDa molecules (phosphotungstic acid) into AC to a depth of 700–800 µm was achieved in 2.5 hours. However, in this study, we delivered MB, a much smaller molecule (~319 Da), into AC at a timeframe of <12 minutes. Therefore, a fair comparison of efficiency of the delivery with the different methods is limited and remains to be investigated.

LU safety in AC was also confirmed by analyzing LU-exposed AC samples for chondrocyte gene expression levels and cell viability. As demonstrated by the results, chondrocyte viability was unaltered by LU exposure following a 24 h period. This results indicates that the short-term LU exposure most likely does not induce cytotoxicity stress in the resident chondrocytes compared to long-term low intensity ultrasound (LIU)^31^. The observed lack of influence on cell viability is in sharp contrast with previously reported decreased viability caused by HIU shock-wave modalities; however this contrast may be due differences in sound exposure (*e.g*. viability was reported to decrease after 4000 pulses at a higher PRF of 10 Hz) and respective interaction with chondrocytes^32^. We observed no LDH release, an indirect indicator of cell death, after 24 h suggesting that cell viability was not significantly impacted. In addition, as observed in EPI fluorescence microscopy, the viability of chondrocytes in AC exposed to LU did not differ from chondrocyte viability in positive control (*p* = 0.686). It should also be considered that the radiation pressure 7.3 kPa is small, which would lead to a fraction of the stress at a fraction of time AC can experience physiologically (from hundreds of kPa’s up to several MPa’s)^33, 34^, therefore subjecting chondrocytes to relatively low mechanical load.

LU exposure led to no significant changes in the studied gene mRNA expression levels. While not statistically significant, a minor, but simultaneous average change was observed in expression of several OA catabolic markers such as *MMP1*, *MMP3* and *MMP9* which points to a possible anti-catabolic effect, which would be in line with low- intensity ultrasound studies^35–37^. Taken together, our results demonstrate that LU did not negatively influence the key essential genes that sensibly balance mechanisms of AC structure, composition, and homeostasis^38^. This further suggests biological short-term safety of LU as a means for agent delivery. In addition, interaction between LU pulses and chondrocytes should further explored if RNA expression levels could be managed by LU in AC.

The attractiveness of LU agent delivery in the context of OA lies in several technical aspects. While the proposed technique could be applied transcutaneously by focusing the sound beam with *e.g*. parabolic reflectors^39^, the technology could be miniaturized for intra-articular operation within the narrow joint space. The optical energy for sound production could possibly be delivered into a joint using a thin (~125 µm) intra-articular optical fiber and apply miniature optical systems to focus the optical beam for plasma generation. Our previous study^20^, we demonstrated that MB delivery using LU can be localized to a confined area near the sound source, *i.e*. plasma spark, potentially enabling treatment of AC tissue locally. To apply drug delivery in a clinical context, *e*.*g*. during arthroscopy, the rate of delivery could possibly be enhanced by delivering higher ultrasound power by increasing (*i*) laser/sound pulse power (*ii*) PRF and (iii) number of induced pulses per treatment. Since the focus of this study was not to optimize the delivery, but rather to provide evidence on the feasibility of LU delivery of agents into AC, future studies will need to investigate how greater quantities of agents could be delivered or deposited into AC in shorter time with less heating, without compromising safety. Particular attention needs to be paid to studying viability, migration, as well as intermediate and long-term adverse effects on gene expression and osteochondral cells (especially chondrocytes).

We chose MB as the delivered entity, because of its similar molecular weight with current OA drugs such as diacerein (~368 Da) and glucosamine (~179 Da). LU drug delivery effect (delivery rate and/or spatial distribution and quantity of delivered cargo) with drugs with different molecular weights, charge and molecule weight could be different to what has been presented in this study. Potential candidates for cargo that could possibly be delivered into AC by LU could be *e.g*. growth factors, cytokine blockers, corticosteroids and protease inhibitors^18^ or drug carriers such as nano-capsules, nano-rods or nano-bubbles. Forcing a drug into AC by external forces such as acoustic radiation force and acoustic streaming, could induce a longer residence time in AC than drugs diffusing into AC passively. An appropriate pre-defined charge of the delivered entity could be used to enhance delivery speed and prolong drug residence in AC.

The limitations of the study include the following. The sample size is relatively small. In addition, sound reflection from the liquid air interface back towards the sample may have affected the delivery. Moreover, compositional or structural tissue changes and effects on cell apoptosis or viability that were not detected in this study, could actually take place in a longer timeframe exceeding the short timeframe of one day. Intermediate and long-term safety, therefore, remains to be investigated. It should also be noted that the performed content analysis is limited only to collagen and proteoglycan, since they are the main constituents of the AC solid matrix. No conclusions can be drawn on effects of LU on contents of minor AC constituents.

LU-induced localized delivery of agents and enhanced retention of such entities could reveal unforeseen scenarios important for development of new strategies for OA drug therapy; one great advantage would be enabling targeted drug therapies that minimize systemic toxicity and that increase the tissue specificity of the treatment.

To conclude, we demonstrated that LU can deliver MB into AC without detected major short-term negative effects on RNA expression, cell viability, structure, collagen content or proteoglycan content. The results point towards exploring new possibilities in localized deposition of drugs into AC using LU.

## Methods

### Samples

Intact bovine stifle joints (pre-defined criteria) (*N* = 16, ≤6 days *post mortem*), one joint per animal, were obtained from a local meat refinery (Lihakonttori Oy, Helsinki, Finland) for drug delivery, as well as for structural and DD assessment. Intact bovine stifle joints (*N* = 10), one joint per animal, were obtained fresh from a local meat refinery (Veijo Votkin Oy, Helsinki, Finland) within 60 hours *post mortem* for mRNA, biochemical and viability assessment. AC in all OC samples (*N* = 26) were visually evaluated to be normal and to have achieved skeletal maturity. The joints were dissected and osteochondral cores (Ø = 13 mm) were drilled from the femoral condyle with a hollow bit and detached with a saw. Subchondral bone was trimmed with a low speed saw (saw: Buehler Isomet, 11-1180-250; diamond blade: 11–4256, Buehler) leaving 1–3 mm subchondral bone beneath AC. Cylinders (*N* = 21 or *N* = 5) were then split into four quadrants (*n* = 84) or two halves (*n* = 10), respectively (Fig. 1). The samples for agent delivery tests (*n* = 40), as well as for structural, DD and FTIRI assessment (*n* = 24), were stored at −16 °C until experiments in room temperature. The halves (*n* = 10) were immediately subjected to LU or control treatment and to subsequent mRNA and biochemical analyses. The quadrants (*n* = 15) were subjected dead-live assessment by fluorescence microscopy and remaining quadrants (*n* = 5) remained unused. All AC explants were frequently rinsed with PBS during sample preparation to maintain AC hydration.

### Group assignment

*For drug delivery experiments*, 4 quadrants from each of 10 osteochondral cylinders were randomly assigned to four statistically dependent groups (Table 1, Fig. 1A): (i) treatment group T1 (simultaneous LU + contrast agent exposure; *n* = 10); (ii) control group C1 (pre-treatment with LU followed by immersion in contrast agent; *n* = 10); (iii) control group C2 (no LU, only contrast agent exposure; *n* = 10); and (iv) control group C3 (no LU or contrast agent exposure; *n* = 10). Immediately after the experiment, AC tissue was detached from the subchondral bone with a scalpel and the samples were frozen to halt further diffusion of MB until cryomicrotomy.

*For structural, DD and FTIRI assessment*, 4 quadrants from each of 6 osteochondral cylinders were randomly assigned to 4 statistically dependent groups (Fig. 1B, Table 1): treatment groups ST1 (*n* = 6) and ST2 (*n* = 6); and control groups SC1 (*n* = 6) and SC2 (*n* = 6). Samples ST1 and ST2 were subjected to LU in PBS immersion, while control samples SC1 and SC2 were not exposed to LU. The groups ST1 and SC1 underwent field emission scanning electron microscopy, whereas ST2 and SC2 were subjected to standard histology, DD and FTIRI assessment as described later.

*For mRNA and biochemical assessment*, the sample halves from 5 osteochondral cylinders were randomly assigned to 2 statistically dependent groups (Fig. 1C, Table 1): treatment group BT (*n* = 5) and control group BC (*n* = 5). Samples BT were subjected to LU in PBS immersion, while control samples BC were not exposed to LU. Both groups underwent mRNA expression analysis and biochemical assessment as described later.

*For viability assessment using EPI fluorescence microscopy*, 3 quadrants from each of 5 osteochondral cylinders were randomly assigned to 3 statistically dependent groups (Fig. 1D, Table 1): treatment group VT (*N* = 5), negative control group VC− (*N* = 5) and positive control group VC+ (*N* = 5). Samples VT were subjected to LU in PBS immersion, while control samples VC− and VC+ were not exposed to LU. Group VC+ was incubated in DMEM for 10 min at +63 °C to induce cell death in chondrocytes. All groups underwent cell assessment using EPI fluorescence microscopy and image analysis as described later.

### LU exposure and contrast agent

LU pulses (*f*_c_ = 3.0 ± 0.1 MHz; bandwidth at −3 dB = 2.5 ± 0.1 MHz; *I*_TP_ = 6 017 ± 188 W/cm^2^; *I*_PA_ = 1 076 ± 90 W/cm^2^; *I*_TA_ = 1.86 ± 0.04 mW/cm^2^; peak-positive-pressure = 9.14 ± 0.15 MPa; peak-negative-pressure = 1.90 ± 0.05 MPa; mechanical index = 1.01 ± 0.03; mean ± S.E.M, *n* = 100; parameters defined at 3 mm distance from the plasma spark) were produced by laser-induced plasma sparks by focusing (*f* = 75 mm) a horizontally aligned laser beam (λ = 1064 nm, 2000 pulses, pulse duration = 8 ns, PRF = 3 Hz, Q-switch delay = 300 µs, pulse energy = 130 ± 10 mJ, treatment time = 11.1 min; Quantel CFR Big Sky Laser Series, Les Ulis, France) 3 mm above AC surface (parallel to the laser beam) (Fig. 2). A treatment consisted of 2000 sound pulses delivered at 3 Hz pulse repetition frequency yielding a treatment time of 11.1 min. The LU parameters were selected based on the following reasoning: Sufficient energy to generate a plasma spark, low PRF (3 Hz) and pulse number (2000) in order to avoid excess heating while creating visible MB delivery.

Depending on group assignment (Table 1) the sample was subjected to (i) LU exposure or (ii) no LU exposure, while immersed in (i) contrast agent solution, *i.e*. 0.005% w/v methylene blue (MB) in PBS, or (ii) PBS only. MB was considered to be a suitable contrast agent, because it strongly absorbs light the visible range (peak absorption at 665 nm) and its molecular weight ~320 Da is close to that of OA drugs such as glucosamine (~179 Da) and diacerein (~368 Da). The total immersion time in MB for samples T1, C1, and C2 was 17.8 ± 2.9 min (mean ± 95% CI, *n* = 19). The time range (17.8 min vs 11.1 min) is due to sample positioning and laser beam alignment. Temperature rise during LU exposure was small, *i.e*. 0.95 ± 0.35 °C (mean ± 95% CI; *n* = 21, baseline: 21.57 ± 0.89 °C). Samples T1, C1, and C2, all from the same joint, were immersed simultaneously to avoid bias across dependent samples.

Following the treatment of BT (LU exposure) and BC (control), explants were cultured in Gibco Dulbecco’s modified Eagle’s medium (DMEM/F-12, Life Technologies, Carlsbad, CA) supplemented with 10% fetal calf serum (FCS) and maintained for 24 h at 37 °C in a humidified atmosphere with 5% CO_2_. After 24 h AC samples and conditioned medium were collected, snap frozen in liquid nitrogen and maintained in −80 °C until further analysis.

### Detection of delivery

The MB concentration was optically detected as follows. The frozen AC sample was moved into a cryomicrotome (Leica CM3050 S, Leica Biosystems, Nussloch, Germany), processed to 150 ± 20 µm sections and placed on a 1 mm thick glass slide. Sections were imaged with a light microscope (LM) (Stemi 2000-C stereo microscope, Oberkochen, Germany) and a Thorlabs camera (1.3 megapixels, model: DCC1645C-HQ, Newton, NJ) in through-transmission mode. The peak wavelength (657 nm) of the transmitted light (light source: part number 148 LXZ1 – PA01, Philips Lumileds Lighting Company, CA, USA) matched the MB absorption peak at 665 nm. The intensity image $I$ of the AC section on a glass slide was divided point-by-point by the reference image ${I}_{0}$ through glass only (Supplementary Figure A1). A region of interest (400 × 1280 pixels) corresponding to 0.7 × 2.5 mm^2^ was used for further analyses. The AC surface was then manually segmented and straightened. The previous was repeated for 3 adjacent AC slices from the same sample and these three images were averaged. The resulting image was horizontally averaged, the depth-wise averaged across 100 µm distance at 100 µm increments, *i.e*. 1–100, 100–200, 200–300 µm etc. to obtain one $\frac{I}{{I}_{0}}$ value for each depth 100–200, 200–300 µm etc. (Supplementary Figure A1). For these depths, an exponential attenuation law was applied to determine the Napierian optical absorbance image *A* (Figure A1): $A=-\mathrm{ln}(\frac{I}{{I}_{0}})$. Absorbance values at different depths in C3 were subtracted from the depth-wise absorbances of T1, C1, and C2 values to eliminate the contribution of AC tissue to absorbance. After this correction, the absorbance values of T1, C1, and C2 were subjected to statistical analyses.

### Structural assessment, DD and FTIRI

To detect potential ultrasound-induced damage in AC tissue, FESEM of AC surface, standard histology (surface to deep tissue sections), and assessment of fixed charge density were applied as follows.

After the experiments, treated (ST1) and control (SC1) samples (Fig. 1) were fixed in formalin for at least 5 days. Next, the samples were dehydrated in an ethanol series with ascending concentration (70%, 80%, 90%, 96%, and 100%). The samples were then treated with hexamethyldisilazane followed by air drying. Finally, samples were glued to a sample holder with carbon glue, coated with a 15 nm thick platinum layer (Agar High Resolution Sputter Coater, Agar Scientific, Essex, UK) and eventually subjected to FESEM (Zeiss Sigma, Carl Zeiss Microscopy GmbH, Germany) imaging with magnifications ×200 and ×1000 with 5 kV and 5 mm working distance. The acquired images were subjected to a blinded reader, who visually evaluated the samples for potential damage (*e.g*. grooves and cracks) and compared the superficial AC morphology of a coded sample to the adjacent coded sample. Following the blinded evaluation, the blinding was uncovered and the groups ST1 and SC1 were compared for differences in AC surface morphology.

For histological assessment, treated (ST2) and control (SC2) samples were fixed in formalin for at least 5 days. Next, the samples were then decalcified with ethylenediaminetetraacetic acid (EDTA). Then the samples were embedded in paraffin, sectioned (3 µm slices), and stained with Safranin O. To evaluate potential ultrasound-induced damage to the AC, the excisions were optically imaged at 1× using a microscope slide scanner (Pathscan Enabler IV, Meyer Instruments, Houston, TX, USA) and at 40× using a LM (Aristoplan, Ernst Leitz Wetzlar, Wetzlar, Germany) and a camera (MicroPublisher 5.0 RTV, Qimaging, Surrey, BC, Canada). The acquired images were subjected to a blinded reader, who visually evaluated the samples for potential damage (*e.g*. potential fibrillation, grooves, and cracks) and compared the AC morphology of a coded sample to the adjacent coded sample. Following the blinded evaluation, the blinding was uncovered and groups ST2 and SC2 were compared for differences in AC morphology.

To quantify fixed charge density (FCD, mainly associated with proteoglycan content), tissue sections were subjected to DD^40, 41^ using a LM (Carl Zeiss Axio Scope-A1, Göttingen, Germany) equipped with a bandpass filter (492 ± 5 nm) and a 12-bit CCD camera (Retiga 4000 R, Qimaging, Surrey, BC, Canada). The AC surface was imaged at 40x in transmission mode and images were calibrated against neutral density filters (Edmund Optics Ltd., Nether Poppleton York, UK) with optical density (OD) ranging from 0 to 3 OD.

To quantify collagen content, unstained histological sections from treated (ST2) and control (SC2) were immersed in 1000 U/ml hyaluronidase (Sigma-Aldrich, H3506) in 0.1 M PBS (pH = 6.9, temperature = 37 °C) for 18 h to remove proteoglycans and placed on 0.5 mm thick Zinc-Selenide windows (Crystran Ltd., Poole, UK). FTIR spectra were collected with Nicolet iN10 MX (Thermo Fisher Scientific Inc., Waltham, MA; imaging settings: 10 × 10 μm^2^ pixel size, 8 cm^−1^ spectral resolution and 1 s acquisitions per pixel). Depth-wise collagen distribution was determined from maps representing Amide I absorption (integrated area 1590–1720 cm^−1^) of the spectrum^42, 43^.

### Cell viability

The toxicity of LU treatment on the viability of chondrocytes within the AC explants was determined after 24 h by colorimetric assay of lactate dehydrogenase (LDH) activity in the culture media. Conditioned media was assessed by screening for the production of LDH using the Cytotoxicity Detection Kit (Roche, Penzberg, Germany), according to manufacturer’s instructions and by comparing with the conditioned media from control experiments. Absorbance values at 492 nm were measured using a spectrophotometric plate reader (FLUOstar Omega, BMG Labtech GmbH, Offenburg, Germany), with background media absorbance values subtracted.

To qualitatively confirm the immediate effect of LU treatment on chondrocyte viability, negative and positive controls and LU-treated bovine cartilage explant disks were cut into ~500 μm thick slices (cross-sections from surface-to-deep). 6.57 μg/ml of calcein acetoxymethyl (AM) (C3099, Thermo Fischer Scientific Inc.) in DMEM was used to stain viable cells green, while in 40.06 μg/ml of propidium iodide (P3566, Thermo Fischer Scientific Inc) in the same solution stained the nuclei of dead cells red. Negative controls were non-sonicated and treatment samples were sonicated as described earlier. Cross-sections slices were stained for 30 minutes in the dark at +4 °C and then washed twice in PBS. Cross-section slice were visualized with EPI fluorescence microscope (Leica DM6000 B/M; Leica Microsystems GmbH, Wetzlar, Germany). Live cells were visualized using a 5× objective under a calcein-equivalent (λ_ex_ ~480 nm, λ_em_ ~527 nm) filter and dead cells were visualized under a PI-equivalent (λ_ex_ ~560 nm, λ_em_ ~645 nm) filter. The images were recorded using a Leica DFC 420 digital camera using Leica Application Suite version 3.8.0 software running on a PC. Live and dead chondrocytes were counted (automated Cell Counter plugin, imageJ v1.51 g) and number of viable chondrocytes calculated within three 500 × 100 µm regions-of-interest (ROI) at superficial, middle and deep cartilage. The viability (%) within the sample was presented as an average of the detected viabilities (%) in the ROIs.

### RNA extraction

As high upregulation of catabolic players is prominent during AC degeneration and OA progression, we studied whether LU could alter their baseline expression on healthy AC and, therefore, alter their metabolism. AC explants were placed in a TRIzol lysis buffer (Invitrogen) and lysed according to a Trizol protocol^44^. This was followed by column purification with the RNeasy kit (Qiagen) according to the manufacturer’s instructions. RNA concentrations were measured using a NanoDrop ND-1000 instrument (Thermo Fisher Scientific, Waltham, MA). The RNA integrity number (RIN) and 28 s/18 s ratio were estimated using the RNA 6000 Nano Assays on an Agilent 2100 Bioanalyzer (Agilent Technologies, CA). The cDNA synthesis was performed using approximately 1–2 μg of total RNA and the iScript™cDNA Synthesis Kit (Bio-Rad Laboratories, Hercules, USA) in a 20 μl reaction volume.

### Droplet digital polymerase chain reaction (ddPCR)

Absolute expression levels were measured by droplet digital PCR performed using a Bio-Rad QX200 Droplet Digital PCR system (Bio-Rad). Reactions were performed in appropriate volumes using 10 μl ddPCR EvaGreen SuperMix, 2 μl of target gene primer, 8 μl nuclease free water, and 1 μl cDNA sample, following manufacturer instructions. Samples were loaded into a droplet generator cartridge. Preparation samples (20 μl) were transferred into the supplied cartridges. Droplet generation oil (70 μl) was added into parallel wells of the samples cartridge followed by droplet generation procedure (Bio-Rad). Once the process was complete, droplets (40 μl) were transferred into columns of a 96-well PCR plate and sealed with a supplied pierceable foil in the PX1™ PCR Plate Sealer instrument (Bio-Rad). The sealed plate was loaded into a T100 Thermal Cycler (Bio-Rad). The following PCR program was run: 95 °C for 10 min, followed by 40 cycles first at 94 °C for 30 s, then at 60 °C for 1 min, finally at 98 °C for 10 min. After PCR was complete, the sealed plate was loaded into the droplet reader for detection of completed PCR reactions in individual droplets. The data was visualized and analyzed using the QuantaSoft software v1.7 (Bio-Rad), which measured the fraction of positive droplets and calculated the amount of template per droplet based on a Poisson distribution (includes precision estimates a 95% confidence interval (CI) for each droplet). Thresholds for detection were set manually based on results from negative control wells containing water instead of RNA. Primer sequences are provided in Table A1 (Supplement).

### Statistical analyses

Sample size (*n* = 5–10 per group) permitted the following statistical analyses: The groups T1, C1, and C2 were compared at each depth using a non-parametric Friedman test followed by a pair-wise non-parametric Wilcoxon signed-rank post-hoc test for 2 related samples (SPSS v22.0.0.0, Chicago, IL, USA). The groups VT, VC−, and VC+ were compared using a non-parametric Friedman test followed by a pair-wise non-parametric Wilcoxon signed-rank post-hoc test for 2 related samples. Comparison of sample group pairs BT vs. BC were subjected to non-parametric Wilcoxon signed-rank test for 2 related samples.

## Electronic supplementary material

Supplementary information
