# Supplementary material for: Laser-ultrasonic delivery of agents into articular cartilage
Source: Sci Rep. 2017 Jun 21;7:3991. doi: 10.1038/s41598-017-04293-5 (PMC5479804; doi:10.1038/s41598-017-04293-5)
Supplement: Supplementary file 1 — Supplementary information [file 41598_2017_4293_MOESM1_ESM.pdf]

Supplementary material to manuscript:

Laser-ultrasonic delivery of agents into articular cartilage

Heikki J. Nieminen, Gonçalo Barreto, Mikko A. Finnilä, Alejandro García-Pérez, Ari Salmi, Sanjeev Ranjan, Kari K. Eklund, Kenneth P.H. Pritzker, Simo Saarakkala, Edward Hæggström

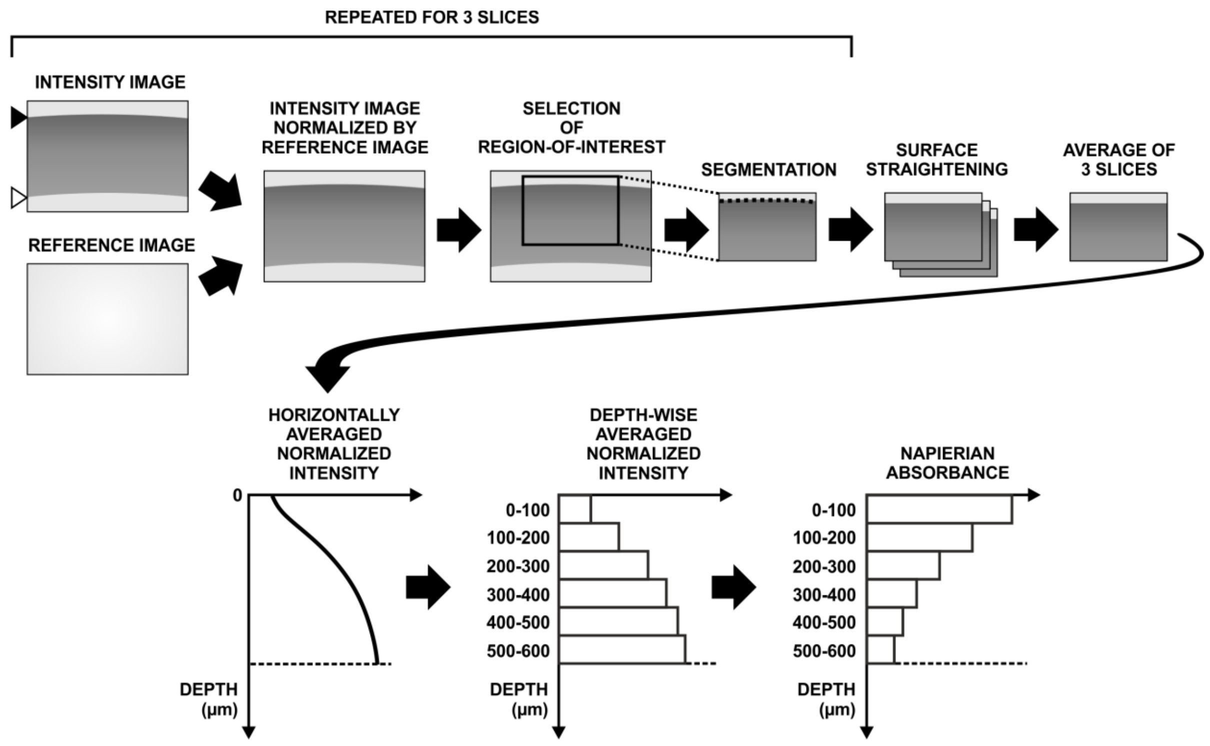

Figure A1: *The delivery of methylene blue (MB) was detected from light microscope images acquired in through transmission mode (light wavelength λ = 657 nm). Napierian optical absorbance image was calculated based on optical intensity images acquired from 3 articular cartilage sections per sample (with microscope glass) and a reference images (microscope glass only). Depth-wise absorbance was determined at depths 0-100, 100-200, 200-300 µm etc. Absorbance is linearly associated with concentration of light-absorbing medium and is, therefore, used as an indicator for MB content in the tissue.*

TABLE A1. *Primer pairs used for droplet digital polymerase chain reactions.*

Gene	Protein name	Primer Sequence
ACAN	Aggrecan	Forward: 5 ´ - CGA TAC CCC ATC TCC AAG GC- 3′
		Reverse 3 ´ - AGT GAT ATT TCG GGG CAG GG - 5′
COL2A1	Collagen, type II, alpha 1	Forward: 5 ´ -ACT GCC AGT GGG CAA CCA - 3′
		Reverse 3´- GTC ACC ACG ATC ACC TCT GG - 5′
MMP1	Matrix metalloproteinase 1 (MMP-1)	Forward: 5 ´ - AAA TCC CAC TCA GCC AGT CG - 3′
		Reverse 3´- CCC TGT CGG CAA CCT CAT AA - 5′
MMP3	Matrix metalloproteinase 3 (MMP-1)	Forward: 5 ´ - CAC TCA ACC GAA CGT GAA GC - 3′
		Reverse 3´- GCT GAC TGC ATC GAA GGA CA - 5′
MMP9	Matrix metalloproteinase 9 (MMP-9)	Forward: 5 ´ - GAG ATG CCC ACT TCG ACG AT - 3′
		Reverse 3´- GAG CGA CCC TCA AAG GTG AA - 5′
MMP13	Matrix metalloproteinase 13 (MMP-13)	Forward: 5´- GTT GCT GCC CAT GAG TTT GG - 3′
		Reverse 3´- TGT CTG GCG TTT TGG GAT GT - 5′
TGFB	Transforming Growth Factor Beta	Forward: 5´- AAT AGA GGG CTT TCG CCT CAG - 3′
		Reverse 3´- AGC AGT AGT TGG TGT CCA GG - 5′
TIMP3	Tissue Inhibitor Of Metalloproteinases 3	Forward: 5´- GGC CGA GTC TAT GAT GGC AA - 3′
		Reverse 3´- GTT TGG ACT GGT AGC CAG GG - 5′
TNF	Tumor necrosis factor	Forward: 5´- GAA GAG CAG TCC CCA GGT G - 3′
		Reverse 3´- GGG CTA CCG GCT TGT TAC TT - 5′
